# The Construction and Validation of the Heat Vulnerability Index, a Review

**DOI:** 10.3390/ijerph120707220

**Published:** 2015-06-26

**Authors:** Junzhe Bao, Xudong Li, Chuanhua Yu

**Affiliations:** 1 School of Public Health, Wuhan University, 115 Donghu Road, Wuhan 430071, China; E-Mail: junzhe_bao@126.com; 2 Office of Epidemiology, Chinese Center for Disease Control and Prevention, 155 Changbai Road, Changping District, Beijing 102206, China; 3 Global Health Institute, Wuhan University, 115 Donghu Road, Wuhan 430071, China

**Keywords:** extreme heat, heat vulnerability index, vulnerability map, validation of HVI

## Abstract

The occurrence of extreme heat and its adverse effects will be exacerbated with the trend of global warming. An increasing number of researchers have been working on aggregating multiple heat-related indicators to create composite indices for heat vulnerability assessments and have visualized the vulnerability through geographic information systems to provide references for reducing the adverse effects of extreme heat more effectively. This review includes 15 studies concerning heat vulnerability assessment. We have studied the indicators utilized and the methods adopted in these studies for the construction of the heat vulnerability index (HVI) and then further reviewed some of the studies that validated the HVI. We concluded that the HVI is useful for targeting the intervention of heat risk, and that heat-related health outcomes could be used to validate and optimize the HVI. In the future, more studies should be conducted to provide references for the selection of heat-related indicators and the determination of weight values of these indicators in the development of the HVI. Studies concerning the application of the HVI are also needed.

## 1. Introduction

It is well known that the global climate will become warmer in the future, and many researchers have estimated that the occurrence of heat waves and their adverse effects will be exacerbated by this increase in global temperature [1,2]. Heat waves can result in high numbers of heat-related illnesses and deaths. In 1980, a heat wave killed approximately 10,000 people across the United States [3]. In July of 1995, a heat wave also killed more than 700 people in Chicago [4], and in August of 2003, a heat wave killed more than 70,000 people in Europe [5]. Furthermore, extreme heat has been found to be the deadliest weather-related hazard in some locations [6,7]. People with respiratory or cardio-vascular diseases, diabetes, chronic mental disorders or other pre-existing medical conditions are at greatest risk of being negatively affected by heat waves [8,9]. Individuals who are elderly, are socially isolated, have a low income, are uneducated, or live in low-income housing are also at greater risk [10,11].

The adverse health effects that result from extreme heat can be prevented. Some cities that implement hot weather response measures during heat waves have experienced decreases in the morbidity and mortality of heat-related illnesses [12,13]. A review of emergency response plans found that half of the studied cities had specific plans for extreme heat events but that few cities had interventions to reach the at-risk populations [14,15]. To more effectively prevent heat hazards, it is important to determine both the regions and those people within the regions who are at an increased risk of being negatively affected by heat waves [16,17,18].

Vulnerability typically refers to the capacity to be wounded, e.g., the degree to which a system is likely to experience harm due to exposure to a hazard [19]. In the field of climate change, vulnerability is the degree to which a system is susceptible to, or unable to cope with, the adverse effects of climate variability and change. From a health perspective, vulnerability can be defined as the summation of all risk and protective factors that ultimately determine whether a subpopulation or region experiences adverse health outcomes due to climate change. Vulnerability is a function of the character, magnitude, and rate of climate change as well as the variation to which a system is exposed, its sensitivity, and its adaptive capacity [20]. By assessing the heat vulnerability of different places and populations, managers can target resources to the places and populations that are at a higher risk of being affected by extreme heat.

Heat vulnerability can be assessed by some determinants that have heat-related health effects, and these determinants can be determined through studying epidemiologic literature, consulting relevant experts, and referring to related research. The published literature on the vulnerability to heat is substantial [21,22,23] but quantitative assessments are relatively rare, and different methods are typically utilized to determine the final heat vulnerability index (HVI) [24,25]. Therefore, it is essential to compare and review these studies to better understand the construction of the HVI.

To easily compare the vulnerability of different areas to extreme heat and to undertake further spatial analysis, the HVI can be inserted into the map through geographic information systems (GIS). Researchers can also perform further spatial autocorrelation analyses and other spatial analyses through GIS [26,27].

After acquiring the HVI of each research area, the use of health data to validate the effect of the HVI is essential before its application in practice. The health data may include the ambulance callouts and the morbidity and mortality data of heat-related illnesses on abnormally oppressive days [28,29,30].

In this review, we included 15 studies on the HVI, and we analysed the determinants and the methods they adopted in the construction of the HVI. What’s more, we analysed some studies on the validation of the HVI. With this review, we hope to provide some useful references for the future study on the HVI.

## 2. Methods

A literature search was conducted to identify articles concerning the HVI published up to 30 January 2015. Several online databases were queried, including Web of Science, Science Direct, PubMed, and Google Academic. The following key words were used individually and in combination as inclusion criteria for articles to be considered for this review: heat, hot, high temperature, thermal, vulnerability, risk, resilience, index (indices), geography, and GIS. Peer-reviewed, English-language journal articles were included. References and citations of the relevant articles were inspected manually to ensure that all relevant articles were included. Eligibility included any studies that quantitatively assessed the health vulnerability to oppressive heat, especially those regarding the HVI.

**Figure 1:**
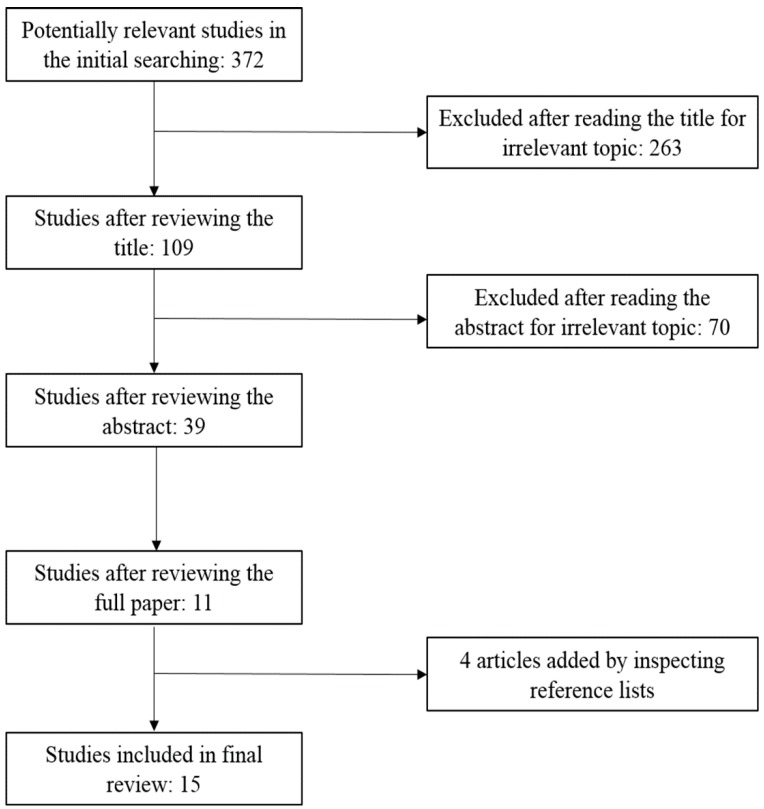
The literature selection process.

## 3. Results and Discussion

We include 15 studies in this review (Figure 1), and the characteristics of the 15 studies are summarized in Table 1, which includes the author, year of publication, study location, selected determinants and main methods used in the studies.

**Table 1:** Summary of the studies about heat vulnerability assessment.

Author (Time, Location)	Variables (Numbers)	Methods
Vescovi *et al.* [31] (2005, Southern Quebec)	hot days, consecutive hot days with Tmax > 30°C and Tmin > 22°C, elderly, poverty, isolation, education (6)	normalization, equal weight
Reid *et al.* [25] (2009, USA)	poverty, education, ethnicity, living alone, elderly, aged people living alone, vegetation, diabetes, central AC, AC (10)	principal component analysis
Rinner *et al.* [24] (2010, Toronto)	land surface temperature, vegetation, old dwelling without AC, high-density dwellings without AC, behavior, illness, cognitive impairment, elderly, infants and young children, poverty, rental households, isolation, homeless, education, not English speaking, recent immigrants, ethnicity, home cooling, drop-in centers, participating community outreach centers, cooling centers (21)	ordered weighted averaging, local indicators of spatial association
Hondula *et al.* [32] (2012, Philadelphia)	surface temperature (2004 and 2008), low/mid/high density residential, recreational, industrial, mixed use land, commercial, building coverage, White, Black, American Indian, Asian, Pacific Islander, other race, two or more races, nonwhite, elderly, education, income, below poverty line, below 2x poverty line, aged people living alone, living alone(25)	principal components analysis, multiple linear regression
Chow *et al.* [21] (2012, Phoenix)	mean summer maximum/minimum temperature, mean normalized difference vegetation index, elderly, income, foreign-born noncitizens, immigrants (7)	normalization, equal weight
Tomlinson *et al.* [33] (2012, Birmingham)	land surface temperature, elderly, ill, density of households, flat (5)	normalization, equal weight
Loughnan *et al.* [34] (2012, Melbourne)	aged care facilities, ethnicity, aged people living alone, infants and elderly, urban density (5)	linear correlation, regression analysis, weighting the indicators according to their contribution
Johnson *et al.* [35] (2012, Chicago)	land surface temperature, elderly women, elderly men, lonely elderly women, white population, female heads of household, lonely elderly men, family income, per capita income, household income, population with less than high school education, Asian population, population aged 65 and older in group living, other race population, Hispanic population, population 25 and older with a high school education, built-up index, vegetation index, black population (19)	principal component analysis
Wolf *et al.* [26] (2013, London)	land surface temperature, households in rented tenure, flat, population density, households without central heating, elderly, self-report bad health status, receiving social benefit, single pensioner households, ethnicity (10)	principal component analysis, spatial clustering analysis
Aubrecht *et al.* [36] (2013, U.S. National Capital Region)	heat wave day count, elderly, living alone, poverty, poor English skills, education, vegetation (7)	normalization, equal weight
Harlan *et al.* [37] (2013, Maricopa county)	ethnicity, immigrant, poverty, education, central AC, elderly, elderly and living alone, living alone, unvegetated area (mean), unvegetated area (SD), surface temperature (11)	principal component analysis, local indicators of spatial association
Maier *et al.* [28] (2013, Georgia)	poverty, education, ethnicity, living alone, elderly, elderly and living alone, diabetes, land use (8)	principal component analysis
Dong *et al.* [38] (2014, Beijing)	heat wave days, extremely high temperature days, population density, elderly ratio, income level, land use/cover (6)	normalization, equal weight
Zhu *et al.* [39] (2014, Guangdong)	elderly, infant, immigrant, unemployment, agricultural population, infant mortality rate, health worker, GDP per capita, living space, harmless sanitary latrines, illiteracy rate, temperature growth, heat wave day count (13)	analytic hierarchy process, principal component analysis
El-Zein *et al.* [40] (2015, Sydney)	maximum temperature, minimum temperature, high temperature days, land cover, population density, road density, elderly, elderly and living alone, children, multiunit dwellings, population completing year 12, not English speaking, home loan repayment, home ownership, household income, internet access, assets to liabilities of local council, business rates, residential rates, community service expenses, environmental and health expenses, population requiring financial assistance (22)	multi-criteria outranking approach

### 3.1. Heat Vulnerability Determinants

Romero-Lankao *et al*. [41] utilized a meta-analysis and meta-knowledge approach to study the urban vulnerability to temperature-related hazards and found that this relationship had mostly been examined using 13 factors: hazard magnitude (*i.e.*, temperature level), population density, age, gender, pre-existing medical conditions, education, income, poverty, minority status (African American, Non-African American minorities, and non-white, these terms only make sense in the U.S.), acclimatization, and access to home amenities such as air conditioning and swimming pools. These drivers accounted for 66% of the total tallies of vulnerability determinants. Based on the levels of evidence and agreement, they studied these determinants (Table 2).

Using the Generalized Linear and Mixed Model, Uejio *et al.* [42] derived the relative importance of heat exposure, socioeconomic vulnerability, and neighborhood stability from locations where people suffered from heat distress or mortality. They studied US two locations, Philadelphia (PA) and Phoenix (AZ). They found that in Philadelphia, the final influencing factors were as follows: African-American, the year the house was built, vacant households, and total population. In Phoenix, the final influencing factors were night-time surface temperature, impervious surface, housing density, households renting, population aged 65 and older, people living alone, linguistically isolated households, Hispanic, Asian ethnicity, vacant households, imperviousness surface x housing density, and total population.

**Table 2:** Determinants of heat vulnerability, levels of evidence and agreement [41].

Amount of Evidence	Large	Gender: Female	Age (+) Education (−)	Magnitude (+)
	medium	income race: Non-African American minorities	population density (+) poverty (+) deprivation (+) housing quality (~) social isolation (~)	timing (+) pre-existing medicalconditions (+) acclimatization (−) race: African American (+) air conditioning (−)
	small	housing density social networks	total population (~) urban land use (+) open space (~) vegetation (−) healthcare access (−)	duration (+) variance (+) race: non-white (~)
		low	medium	high
		level of agreement		

Notes: “Amount of evidence” represents the amount of empirical evidence available in the literature; “Level of agreement” represents the level of agreement across different studies. Symbols in parentheses denote the direction of the relationship between each specific factor and heat vulnerability that was identified in the majority of studies, in cases of medium or high level of agreement only. +, positive relationship (increases vulnerability); –, negative relationship (decreases vulnerability); ~, no relationship.

#### 3.1.1. Thermal Characteristics

Romero-Lankao *et al*. [41] found that hazard magnitude (*i.e.*, temperature level) was the only determinant that had been extensively studied (*i.e.*, has a large amount of evidence) and showed a high level of agreement in its effects across different studies in the field of urban vulnerability to temperature-related hazards (Table 2).

Land surface temperature (LST) is widely used to reflect temperature conditions [24,33,35,43] and is measured using airborne or satellite remote sensing [44], whereas air temperature is measured in the canopy layer (a layer of air aboveground equivalent to the height of proximate buildings and trees) using in situ data from meteorological station networks, automobile transects, or specialized sensor platforms [45]. The number of hot days (the days with the maximum temperature higher than a specific value) or heat wave days (consecutive period of at least specific days during which the daily maximum temperature is higher than or equal to a specific value) was usually used as the exposure indicator in the construction of the HVI [31,36]. Besides, high minimum temperatures and temperature changes are also considered to be influencing factors in heat-related deaths [46,47]. Because that the requisite climate station density for resolving intra-urban variations in air temperature does not exist for many researches, so LST is usually adopted in such studies [26]. Although air and LST can display similar spatial and temporal patterns, micro-scale site characteristics have a greater influence on surface temperature than on air temperature [48]. The relationship between air and surface temperatures is also heavily influenced by weather conditions. In cloudy, windy weather, the effect of surface temperature on air temperature is diminished [49]. Furthermore, air temperature is influenced by humidity, and wind determines the degree of comfort for the human body and directly influences morbidity and mortality during extreme heat events [27,50].

Kershaw *et al*. [51] used the humidex, which is an index developed by Canadian meteorologists, to reflect the “actual feel” of hot and humid weather, to estimate the apparent temperature. The humidex combines air temperature and relative humidity into a single value. Meanwhile, some researchers have used the heat index to reflect the apparent temperature [52,53,54,55,56]. In order to account for local adaptation in each area, Maier *et al*. [28] using 90th and 95th percentiles of both maximum and minimum apparent temperatures for the warm season and by month in the definition of oppressive heat.

#### 3.1.2. Demographic and Socioeconomic Factors

Age can affect how efficiently an individual’s body can adapt to inclement weather and maintain normal thermoregulatory processes. The elderly’s adjustment ability is relatively poor, and they usually have chronic illness [52,57] and a higher probability of social isolation. Therefore, old age increases the risk of adverse effects in the face of extreme heat [12,58]. The following groups are also at increased risk: children, as they physiologically have greater susceptibility to the effects of heat [59,60]; those living in poverty, as they have less of an ability to control, change or mitigate risk situations [16,61]; those living alone, as they are likely to ignore the heat risk and lack support [62]; those who are non-white, as they are more likely to be affected by heat [57,63]; those who do not have access to cooling devices such as air conditioning [61]; those who have some pre-existing medical conditions such as mental disorders, diabetes, cardiovascular and respiratory disease [64,65,66]; and those who live in a location with small green area, as vegetation is often associated with affluence and has a significant and negative correlation with temperature and the occurrence of extreme heat events [67,68].

### 3.2. The Construction of the HVI

After confirming the determinants of heat vulnerability, many researchers have normalized the values of the determinants to relative positions between 0 and 1. Some have assumed that all of the determinants are of equal importance and thus weighted them equally, and the normalized values of all determinants were aggregated to form a composite HVI [30,31,36]. This approach helps avoid additional subjectivity in the index development. Indicator standardization (a 0–1 range) and un-weighted quantitative aggregation (additive approach) are a common approach in indicator composition [69,70]. Alternative strategies include expert judgment [71,72] or multivariate statistical techniques such as principal component and factor or cluster analysis [73].

To limit the number of variables and create independent factors for inclusion in a vulnerability index, most of the related studies included a principal components analysis. A varimax rotation was usually used to minimize the number of the original variables that loaded highly on any one factor and increased the variation among factors, thus making these new factors more statistically independent than the original variables. Furthermore, the eigenvalues, scree test and percentage of variance explained by the factors were usually used in the process of selecting the final analysis factors [25,26,35,37].

Rinner *et al*. used ordered weighted averaging (OWA) in their construction of the HVI. OWA is a family of multi-criteria operators allowing the decision-maker to apply weights of importance to the criteria and to specify an attitude towards decision risk. A decision strategy can be specified mathematically (using an additional set of weights) on a scale from risk-averse (“pessimistic”) to risk-taking (“optimistic”) [24,74].

Loughnan *et al*. [34] studied the association between selected variables and the sum of emergency admissions and deaths (AHO), and two variables that did not show a significant correlation with AHO were not considered further for inclusion in the vulnerability index. They also used a condition index to identify possible collinearity between index variables. Then, a stepwise linear multiple regression between AHO and the remaining variables was calculated. Five of eight variables made significant contributions to the spatial distribution of the vulnerability index. The five weighted values were calculated for each variable in each postal area (POA) in the index, and the weighted variable values were then summed in each POA. Each POA was given a decile rank (relative to all of the other POAs), with the lowest 10% being decile 1 and the highest 10% being decile 10. The ranks for each variable were calculated, and an increase in rank indicated an increase in vulnerability. Their result was subsequently mapped using MapInfo software.

Zhu *et al*. [39] invited nine experts from the fields of public health, meteorology, and social sciences to determine the heat-related indicators and assist in the construction of the HVI. A subjective (analytic hierarchy process) and an objective (principal component analysis) method were employed to determine the weight of each indicator. As a result, the estimated vulnerability of principal component analysis had a similar distribution pattern with that estimated by analytic hierarchy process.

At present, a lot of studies adopt one method in their construction of the HVI, in order to obtain a better HVI, two or more methods could be adopted and compared in the future study, just as the study of Zhu *et al*. [39]. Researchers could adopt the data of heat-related health outcomes to assess the fitting effects of different methods and models, and they could compare the fitting effects of the models after adding or deleting some heat-related indicators to optimize the HVI. What’s more, because of the place-specific of the HVI, we recommend that the selection of heat-related indicators and the weight of them should refer to the local characters and base on the heat-related health outcomes. For example, researchers could use a regression model to relate the heat-related indicators to heat-related health outcomes, significant variables could be adopted in the construction of the HVI, and the corresponding regression coefficients or odds ratios could be referred in the determination of indicators’ weights [32,34].

### 3.3. Validation of the HVI

An inherent danger in developing vulnerability indices is that they become little more than mathematical expressions of an eloquent conceptual model of vulnerability if they are not confronted with observational data and tested. To assess the HVI in terms of its ability to predict whether mortality and ambulance callout attain above average levels during heat wave events, Wolf *et al*. [30] adopted three approaches: (1) calculation of categorical statistics and associated skill scores for the dichotomous situation where either above average mortality or ambulance callout occurred or did not occur; (2) the degree to which the relative risk of the aforementioned health outcomes changed with an increase in heat vulnerability, as established using the Poisson regression analysis; and (3) an independent samples test of the difference of mean mortality and ambulance callout between census units with and without high heat exposure and high vulnerability. Their assessment results revealed that the HVI offered potential as a priori indicator of the level of ambulance callout and mortality for all summer days and heat wave events, respectively.

In their assessment of the performance of the HVI across Georgia (USA), Maier *et al*. [28] used the Poisson mixed effect model with natural splines to execute two objectives: (1) to determine if oppressively hot days, as identified by various meteorological metrics, responded with greater mortality than days that were not oppressively hot; (2) to determine if counties with greater vulnerability, as characterized by the HVI, responded with greater increases in mortality on oppressive days than non-oppressive days compared with counties with lower HVI values. The total mortality was set as the response variable, and the HVI and oppressive heat were set as the predictor variables. As a result, except for the least vulnerable categories, the relative risk of mortality increased with increasing vulnerability. For the highest-vulnerability counties, oppressively hot days led to a 7.7% increase in mortality.

Reid *et al*. [29] studied whether areas with higher heat vulnerability, as characterized by the HVI, experienced higher rates of morbidity and mortality on abnormally hot days. They used Poisson regression to model the interaction of the HVI and deviant days (days whose deviation of maximum temperature from the 30-year normal maximum temperature is at or above the 95th percentile) on hospitalization and mortality counts in five states participating in the Environmental Public Health Tracking Network from 2000 through 2007. They found that the HVI was associated with higher hospitalization and mortality rates in all states on both normal days and deviant days. However, associations were significantly stronger on deviant days for heat-related illness, acute renal failure, electrolyte imbalance, and nephritis in California, heat-related illness in Washington, all-cause mortality in New Mexico, and respiratory hospitalizations in Massachusetts.

Chuang *et al*. [75] predicted hospitalization for heat-related illness with the HVI at the census tract level, and the method they used in the construction of the HVI referred to Reid *et al*. [25], whose method was set for the assessment of the HVI at the national level. They used multinomial logistic regression in their study, with health outcomes as dependent variables and factors extracted from heat-related indicators (factor analysis) as independent variables. As a result, they found that the overall accuracy rate of the HVI in predicting heat-related outcomes was only 54% in their study, suggesting that accounting for additional heat-related indicators beyond those they adopted in the construction of the HVI would improve risk prediction. What’s more, they also found that the scores of the HVI did a better job in predicting non-vulnerable areas than vulnerable areas.

## 4. Conclusions and Outlook

The HVI can be constructed by some heat-related indicators, it could be used to highlight the areas where people are at the greatest risk of harm from heat, and it could assist the heat warning systems and government regulators in targeting these high risk areas and protecting people’ s health more effectively on abnormally oppressive days. Heat-related health outcomes could be used to validate and optimize the HVI.

Given that recent studies involving HVI have some differences in selecting heat-related indicators and usually grant equal weight to these indicators in the process of obtaining the HVI, in the future, more studies should be completed to provide references for the selection of heat-related indicators and for the determination of their weight values in developing the HVI. At present, studies that assessing heat vulnerability are mainly performed in Europe and the United States. Because different places may have different situations, we recommend that more countries and regions perform such assessments. For some data poor areas, the construction of the HVI could refer to the studies we reviewed in this paper, and some common indicators such as temperature level, elderly, poverty, education and so on should be adopted as indicators, and it will be better to adopt some heat-related health outcomes to validate and optimize the HVI they get. Furthermore, studies concerning the application of the HVI are also needed.
